# Supporting sexuality for people living with epidermolysis bullosa: clinical practice guidelines

**DOI:** 10.1186/s13023-020-01640-0

**Published:** 2021-01-06

**Authors:** Alex King, Humphrey Hanley, Mark Popenhagen, Florencia Perez, Kerry Thompson, Diana Purvis, Nora Garcia, Ida Steinlein, Mia Werkentoft, Matthew Lightfoot, Michelle Lahat, Kalsoom Begum, Julio Tanabe

**Affiliations:** 1 Department of Rehabilitation, Phoenix Children’s Hospital, 1919 East Thomas Rd, Phoenix, AZ 85016 USA; 2 DEBRA New Zealand, 123 Daniell Street, Newtown, Wellington, 6021 New Zealand; 3 DEBRA Chile, Francisco de Villagra 392, 7760099 Ñuñoa, Santiago Chile; 4 Royal Adelaide Hospital, Port Road, Adelaide, SA 5000 Australia; 5 Starship Children’s Hospital, Park Road, Grafton, Auckland, 1023 New Zealand; 6 DEBRA Spain, Asociación DEBRA-Piel de Mariopsa, C/ Jacinto Benavente No 12, 29601 Marbella, Málaga Spain; 7 DEBRA Norge, Oslo, Norway; 8 DEBRA Sweden, Stockholm, Sweden; 9 DEBRA UK, The Capitol Building, Oldbury, Bracknell, Berkshire, RG12 8FZ UK; 10 Children’s Hospital of Los Angeles, 4650 Sunset Boulevard, Los Angeles, CA 90027 USA; 11 University Hospitals Birmingham NHS Foundation Trust, Mindelsohn Way, Edgbaston, Birmingham, B15 2GW UK; 12 Douglas, AZ USA

**Keywords:** Epidermolysis bullosa, Sexuality, Intimacy, Sexual health, Sex, Puberty

## Abstract

This article presents evidence-based Clinical Practice Guidelines (CPG) for the provision of healthcare services to address sexuality for people living with epidermolysis bullosa (EB). Currently, a lack of EB-specific research limits these services to sexual health assessment and intervention strategies designed for the general population. Due to the unique challenges of EB, a rare skin-fragility condition causing blistering responses to minor skin trauma and other systemic and secondary complications, condition-specific strategies are needed to support people with EB in achieving valued sexual lifestyles. This CPG represents the work of an international panel comprised of thirteen members including a medical doctor, nurses, psychologists, a social worker, an occupational therapist, and patient population involvement members living with EB. It describes the development of EB-specific recommendations for two primary domains of assessment and intervention related to sexuality: psychosocial and mechanical. Following a rigorous evidence-based guideline development process, this CPG establishes the first internationally actionable clinical practice recommendations for sexuality-related assessment and intervention for this population. Future research priorities are identified. Supplemental materials included provide additional support to clinicians in developing the necessary understanding and skills to promote equity and efficacy in this care domain.

## Background

Epidermolysis bullosa (EB) is a rare genetic skin-fragility condition characterized by chronic blistering responses to minor skin trauma due to impairments at the dermoepidermal junction. EB is often identified at the time of birth and subsequently diagnosed and treated according to genotypic and phenotypic presentation. The four primary EB subtypes (EB simplex, junctional, dystrophic, and Kindler EB) are further subcategorized by other characteristics including but not limited to the involvement of specific body surfaces, scarring patterns, effects on body systems, changes in oral-esophageal and genitourinary structures, and specific genetic testing results [1]. While EB subtypes may differ in how they present over the lifespan, at this time EB is considered chronic and lifelong. Because of the varied and profound effects of EB on daily life, the intersection between EB and functional participation has become an increasing area of focus in the clinical and research community [2].

This guideline investigates sexuality as one such intersection. For the purposes of this guideline, a broad view of the term sexuality will be utilized which should be considered congruent with the World Health Organization’s (WHO) [3] description of “sexual health,” described as a state of “physical, emotional, mental, and social wellbeing in relation to sexuality” including “the possibility of having pleasurable and safe sexual experiences, free of coercion, discrimination, and violence”.

EB-related barriers to sexuality are unique, limiting the generalizability of other sexual health guidelines to the EB population. No current guidelines or standards exist to support these needs of the EB population, leaving this group at risk for significant inequities in care.

## Objective

- To outline the current understanding of the interaction between EB and sexuality.
- To provide preliminary recommendations for assessment and intervention strategies to support valued sexual participation for individuals living with EB.
- To establish future research priorities within this domain.
- To highlight currently available resources to support clinicians in meeting the expectations of these guidelines (see Additional file 1).

### Guideline users and target group

This guideline is intended for use by all members of a multidisciplinary EB team. The guidelines may also be useful for individuals living with EB and their families, carers, partners, and communities. These guidelines can be applied to support services for all persons of all ages diagnosed with any Epidermolysis Bullosa subtype.

### CPG development

#### Stakeholder involvement and peer review

In 2017, DEBRA International consulted with the international EB community and identified the topic of sexuality as a priority area for population-specific Clinical Practice Guidelines (CPGs). This guideline was developed in accordance with the DEBRA Guideline Development Standard (see Additional file 2). The CPG development group consisted of thirteen international members representing eight countries (see Additional file 3). The draft document was circulated to thirteen international reviewers who are experts and/or healthcare professionals in the field, as well as people living with EB (see Additional file 3). Throughout the CPG development process, panel leads liaised with Kattya Mayre-Chilton at Debra International (DI) for methodological support and guidance.

#### PICO generation and literature search

**Fig. 1:**
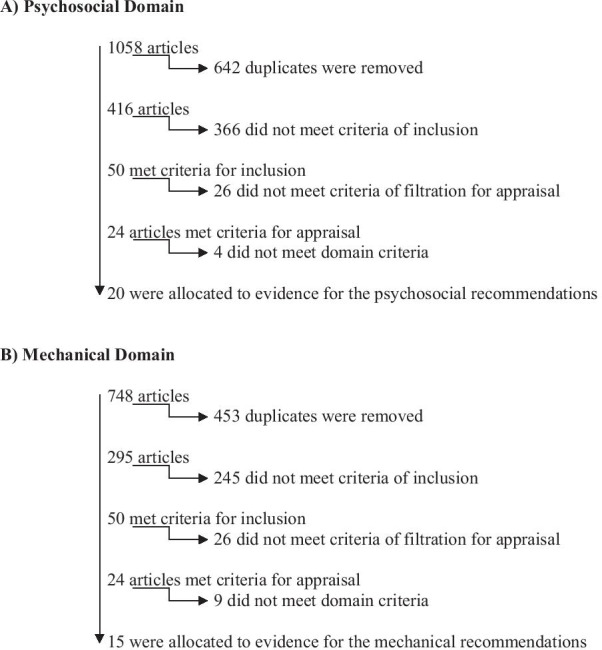
Search results and filtration

From project initiation, the panel consistently emphasized inclusivity of the right to “sexual citizenship” as described by Linton et al. [4] with constant effort to avoid discrimination on the basis of any sexual or personal orientation, preference, age, identity or other demographic. In 2018, the CPG panel confirmed the clinical question: “What sexual health assessment and intervention strategies effectively promote the accessibility of valued sexual participation for people living with EB?” DI and EB-CLINET distributed online scoping surveys developed by the CPG panel. Responses from 63 clinicians and 113 people living with EB (and their families/carers) guided the CPG panel’s focus on two assessment and intervention domains impacting outcomes in sexual health and participation: psychosocial and mechanical (see Additional file 4). Resulting literature search terms and parameters are outlined in Table 1 and Fig. 1.

**Table 1:** Literature search parameters

Databases/engines	Key terms and search
1. EbscoHost 2. PubMed/medline 3. Google scholar Inclusive searches completed from October 2018 to June 2020 *No restrictions* for: Study type Language Interventions *Inclusion Criteria* Human studies only, Relevant to sexual or reproductive health *Exclusion Criteria* If “sex” referred to physiological/biologically male/female participants/gender rather than sexual health/activity	Psychosocial domain Population: Epidermolysis bullosa AND “Sex” OR “Sexuality” OR “Intimacy” OR “Intercourse” OR “Puberty” OR “Sex Education” OR “Body Image” OR “Confidence” OR “Interpersonal Relations” OR “Sexual Behaviour” OR “Health Knowledge Attitudes Practice” OR “Adolescent Behaviour”
	*Mechanical domain* Population: Epidermolysis bullosa AND “Sex” OR “Sexuality” OR “Intimacy” OR “Intercourse” OR “Masturbation” OR “Puberty” OR “Safe Sex” OR “Sex Education”

#### Evidence appraisal for recommendation process

All 24 articles were subject to randomly-assigned systematic quality appraisal by at least two independent panel members to reduce bias. The occupational therapy for EB: CPG [2] modified appraisal tool was utilized. In 2019, the panel produced recommendations using the Grading of Recommendations, Assessment, Development, and Evaluation (GRADE) framework [5] based on the findings of the appraised evidence, expert opinion, and, where indicated, through panel consensus (Table 2). To increase overall strength, a representative cross-section of EB multidisciplinary team specialists (8) and people living with EB (4) peer-reviewed the draft (see Additional file 3), and the Appraisal of Guidelines for Research & Evaluation (AGREE II) tool was conducted by the DI coordinator [6]. The panel addressed all resulting feedback in the final editing stage.

**Table 2:** Recommendations table

Outcome/Recommendation Refer to legend below for clarification of strength and basis of each recommendation	Strength of recommendation	Level of evidence (Range)	Key references
*General panel consensus recommendations*			
(A) Clinicians should evaluate the appropriateness of their role, their clinical skill level, and their personal biases/perceptions related to providing evaluations and interventions recommended in these guidelines and refer for additional supportive services/professionals when needed	D	4	Panel consensus
(B) Clinicians should seek education/training in specific approaches to education and support for sexuality and sexual health	D	4	Panel consensus
(C) Clinicians should utilize established frameworks for introducing and addressing sexuality and pubertal/sexual development when possible	D	4	Panel consensus
(D) Clinicians should ensure knowledge of and adherence to locally relevant professional or governmental restrictions, laws, and requirements regarding the provision of care related to sexuality, including confidentiality rights	D	4	Panel consensus
*OUTCOME: Psychosocial factors impacting sexuality*			
(A) Evaluation of psychosocial factors affecting sexuality			
a. Evaluation should include holistic interview free of influence from clinician values, stigma, or assumptions. Education on clinician role, limits of confidentiality, and patient rights should precede evaluation	C✓	4 to 2++	[7–13]
b. Evaluation should be formative in nature, occurring throughout the lifespan **Families of infants diagnosed with EB should be provided the opportunity for discussion of future sexual participation to minimize assumptions about sexuality-related limitations **Family and child/adolescent readiness for pubertal transition should be assessed in early development **Pubertal stages, timing, and progression should be monitored closely due to risk of pubertal delay in some EB presentations	C✓	4 to 2++	[7–11, 13]
c. Specific and general quality of life measures should be utilized to screen for potential limitations in access to sexual participation **Measures of psychosocial functioning and self-care independence/participation may reveal current or future barriers to sexual participation requiring treatment/referral	C✓	3 to 1+	[12, 14–20]
d. Evaluation should include consideration and/or measurement of vulnerabilities resulting from medical conditions, functional skills, and support needs **Sleep dysfunction, pain, pruritis, energy/strength deficits, and other secondary symptoms/characteristics of EB may significantly impact sexual participation **Functional independence levels in self-care and daily activities may limit access to sexuality-related needs (privacy, hygiene, etc.)	C✓	4 to 1+	[7, 10, 11, 17, 21]
e. General social participation skills and activity levels should be evaluated as a primary component in access to sexual participation	D	4 to 2+	[7–10, 12–15, 19, 21, 22]
(B) Intervention for psychosocial factors affecting sexuality			
a. Clinicians should provide age-appropriate education directly to the individual living with EB throughout the lifespan	D	4 to 2++	[8–11]
b. Clinicians should provide family/carer education during childhood and early adolescence to promote development of autonomy, self-determination, and self-advocacy	C✓	4 to 2++	[7–11, 13, 14, 19]
c. Development of personal identity should be promoted as a primary factor in successful sexual participation. This should include, but not be limited to intervention to improve self-esteem, self-image/body image, sense of belonging, self-confidence, and communication skills for self-advocacy and education of partners/peers **Appearance-related factors in EB can emerge from a broad array of symptoms/factors (bullous formation, scaring, nail changes, keratosis, hair loss, bandaging needs, body weight, etc.). Providing choices in care of these factors may increase treatment relevance to sexual participation and improve perceived control over symptoms/appearance **EB can affect clothing and grooming options significantly. Clothing modification and access to resources to establish a personal “style” or appearance may positively serve psychosocial functioning and mitigate the effect of social stigma for people living with EB	C✓	4 to 1+	[7–17, 19, 21–26]
d. The transition to adolescence should be accompanied by increased privacy, self-determination, and self-care skill development training in the healthcare and health management context	C✓	4 to 2++	[7–9, 11, 14, 23]
*OUTCOME: mechanical factors impacting sexuality*			
(A) Evaluation of mechanical factors			
a. A lifespan approach should be utilized when addressing mechanical factors with evaluation of past, present, and future (desired/anticipated) sexual participation being a standard of care	C✓	4 to 1+	[8, 9, 11, 17, 23, 24, 26–28]
b. Multidisciplinary/Interdisciplinary team is recommended to ensure thorough evaluation of systemic and physical bodily functions that may affect sexual participation	C✓	4 to 1+	[8, 9, 17, 23–28]
c. Early detection and ongoing management of any genitourinary, anal, or oral involvement should be considered a standard of care to promote sexuality **Specific monitoring is recommended for meatal stenosis, genital blistering and/or scarring patterns, microstomia, and dental/oral involvement	D	4 to 1+	[8, 9, 17, 22, 25, 27–29]
d. Formative evaluation of anatomical knowledge, understanding, and self-management skills should be completed throughout the lifespan to promote safe self-exploration and to assess needs for adaptation, training, or further education ** Self-exploration may inform personal sexual preferences, physical needs/limitations, and opportunities for pleasure serving as a primary form of sexual participation, as well as preparation for sexual participation with a partner	D	4 to 2++	[8, 9, 11, 23]
e. Both solitary and interpersonal sexual participation should be considered throughout the lifespan	D	4 to 3	[8, 9, 29, 30]
f. Individualized evaluation/interview regarding valued sexual preferences, activities, and lifestyles should be conducted to ensure education/intervention is applicable and effective for the individual	D	4	[8, 9] Panel consensus
g. Previous and current sexual experiences should be reviewed, including successful and unsuccessful means of physical adaptation	D	4	[8, 9] Panel consensus
h. Assessment of knowledge, understanding, use, and access to sexually transmitted disease prevention and family planning options should be completed prior to intervention	D	4 to 3	[29] panel consensus
(B) Intervention for mechanical factors affecting sexuality			
a. Anatomical, condition-specific, and sexual/pubertal development education to promote safe self-exploration and awareness should be provided at age appropriate levels throughout the lifespan	D	4 to 1+	[8, 9, 11, 17, 23, 26–28]
b. If desired by the individual, masturbation should be addressed as a normal means of self-exploration and sexual participation ** Specific consideration of skin or genitourinary changes, pain, pruritis, or other symptoms resulting from masturbation may indicate need for modification of physical tasks with friction reducing lubrication and/or devices to protect both genitourinary structures and hand structures. Frequency modification may also be indicated	D	4	Panel consensus
c. Interpersonal sexual participation should be considered both possible and natural for people living with EB	D	4 to 3	[8, 9, 29, 30]
d. Mechanical benefits and/or consequences of medical intervention should be considered in the context of sexuality **Gastrostomy tubes, dressings/bandages, and other medical equipment/interventions may have both facilitatory and inhibitory impacts on sexual participation **Activity-specific strategies for protective dressings/bandages, bowel and bladder management, and timing of medications/interventions may improve accessibility of sexual participation	C✓	4 to 1+	[8, 9, 11, 17, 23, 24, 26–28]
e. Anatomical structures valued by the individual for sexual participation should be preserved and/or restored when possible **Surgical and non-surgical treatment of genitourinary, as well as manual, oral, and other physical skills/structures, may increase achievability of valued sexual participation and intimacy	D	4 to 1+	[8, 9, 11, 17, 22, 25, 27–29]
f. Clinicians should provide education and recommendations for means of acquisition of adaptations, modifications, and equipment to reduce friction, improve positioning, and increase comfort and safety in sexual participation **Referral to relevant specialists (occupational therapists, sex therapists, etc.) may be indicated if a person with EB experiences persistent and/or complex mechanical barriers to sexual participation demanding task-specific or contextual adaptation/modification ** If possible, identification of “EB-Friendly” genital lubrication options should be provided to minimize skin trauma and shear/friction during sexual activities	D	4 to 1+	[8, 9, 12, 17, 21, 27, 28]
g. Education for sexually transmitted disease prevention should be provided to all individuals with multidisciplinary team collaboration to optimize options for safety and function	D	4	Panel consensus
h. Education for family planning options should be provided when desired, requested, or required with multidisciplinary team collaboration to optimize options for safety and function	D	4	[29] Panel consensus

Consistent with the occupational therapy for EB: CPG [1], levels of evidence and strength of recommendation grades based on SIGN procedures as delineated in the SIGN50 manual

Levels of Evidence: 4—expert opinion; 3—Non-analytic studies, e.g. case reports, case series; 2−—Case control or cohort studies with a high risk of confounding, bias, or chance and a significant risk that the relationship is not causal; 2+—Well conducted case control or cohort studies with a low risk of confounding, bias, or chance and a moderate probability that the relationship is causal; 2++—High quality systematic reviews of case–control or cohort or studies OR High quality case–control or cohort studies with a very low risk of confounding, bias, or chance and a high probability that the relationship is causal; 1−—Meta analyses, systematic reviews of RCTs, or RCTs with a high risk of bias; 1+—Well conducted meta analyses, systematic reviews of RCTs, or RCTs with a low risk of bias; 1++—High quality meta analyses, systematic reviews of RCTs, or RCTs with a very low risk of bias [31]

Grades for Strength of Recommendations: No A or B present in table; C—A body of evidence including studies rated as 2+, directly applicable to the target population and demonstrating overall consistency of results*; *or Extrapolated evidence from studies rated as 2++; D—Evidence level 3 or 4; or Extrapolated evidence from studies rated as 2+

Indicates that a recommendation achieved panel consensus as a best practice

**Highlights specific considerations based on known natural history of EB supported by evidence and/or panel consensus. This does not represent an exhaustive or universal list of considerations, and individual evaluation remains vital to efficacy of evaluation and care planning

## Results

Responses to scoping surveys directed guideline priorities (see Additional file 4). The recommendation summary has been grouped by outcome domain (psychological and mechanical) with the majority of the articles graded level 3, for small-scale case studies, or level 4, for expert opinion (Table 2). Tables 3 and 4 present a summary of appraised articles and their qualities.

**Table 3:** Overview of evidence for the psychosocial factors impacting sexuality domain

Domains and outcomes	Number of articles allocated	Total participants with EB	Gender Numbers^λ^	Age in years Range	Study type	References
Domain addressing Psychosocial factors impacting sexuality						
(A) Factors such as: a. quality of life b. life span c. stigmas d. vulnerabilities e. access to social participation	16	1153* 700 EB^α^ 143 EBS 121 DDEB 105 RDEB 24 DEB 25 JEB 9 KEB 7 Unknown	83 Male 83 Female	0 to 89	6 Qualitative 4 Systematic literature review 2 Cross sectional observational 2 Symposium report 1 Book chapter 1 Validation study	[7–22] [7, 10, 12, 22]^β^
(B) Interventions such as: a. age-appropriate b. family education c. self-esteem d. body image e. self-advocacy f. transition	20	1301* 852 EB^α^ 168 EBS 122 RDEB 121 DDEB 74 DEB 25 JEB 9 KEB 7 Unknown	97 Male 109 Female	0 to 89	8 Qualitative 4 Systematic literature review 2 Cross sectional observational 2 Symposium report 1 Book chapter 1 Validation study 1 Quantitative 1 Retrospective study	[7–26] [7, 10, 12, 22]^β^

*EBS* EB simplex, *JEB* junctional EB, *DEB* dystrophic EB, *DDEB* dominant dystrophic EB, *RDEB* recessive dystrophic EB, *KEB* kindler EB

*Indicates total number of participants, including those in articles not limited to EB

^α^Indicates total number of participants identified with EB

^β^Indicates articles with participants from a group of conditions including but not limited to EB

^**λ**^Gender numbers represent the sum of demographics reported in the corresponding articles. Please note that very few included articles report gender nor do they indicate gender as self-identified or assigned-at-birth

**Table 4:** Overview of evidence for the mechanical factors impacting sexuality domain

Domains and outcomes	Number of articles allocated	Total participants with EB	Gender Numbers^λ^	Age in years Range	Study type	References
Domain addressing Mechanical factors impacting sexuality						
(A) Mechanical factors: a. across lifespan b. systemic and physical function c. sexual involvement d. self-exploration e. sexual experiences f. sexual knowledge	13	3460 EB^α^ 1688 EBS 457 RDEB 425 DDEB 236 JEB 50 DEB	27 Male 41 Female	1 to 86	3 Systematic literature review 3 Qualitative 2 Symposium report 1 Retrospective study 1 Quantitative 1 Book chapter 1 Registry 1 Case reports	[8, 9, 11, 17, 22–30] [22]^β^
(B) Intervention for mechanical factors affecting sexuality such as: a. condition-specific b. sexual/pubertal c. self-exploration d. medical interventions e. sexual knowledge f. family planning	15	4152* 3692 EB^α^ 1685 EBS 457 RDEB 425 DDEB 236 JEB 50 DEB	27 Male 41 Female	1 to 86	3 Systematic literature review 4 Qualitative 2 Symposium report 1 Retrospective study 1 Quantitative 1 Book chapter 1 Validation study 1 Registry 1 Case reports	[8, 9, 11, 12, 17, 21–30] [12, 22]^β^

*EBS* EB simplex, *JEB* junctional EB, *DEB* dystrophic EB, *DDEB* dominant dystrophic EB, *RDEB* recessive dystrophic EB, *KEB* Kindler EB

*Indicates total number of participants, including those in articles not limited to EB

^α^Indicates total number of participants identified with EB

^β^Indicates articles with participants from a group of conditions including but not limited to EB

^**λ**^Gender numbers represent the sum of demographics reported in the corresponding articles. Please note that very few included articles report gender nor do they indicate gender as self-identified or assigned-at-birth

## Conclusions

While research data on the topic of sexuality and EB is limited, there is enough data for this panel to state the following: A diagnosis of EB does not inherently negate or inhibit an individual’s desire or ability to participate in sexual activities, nor does it negate the human right to expression of an individual’s sexuality. As such individuals living with EB require of the health care team an approach to sexual health which addresses all of the factors relevant to the general population, as well as EB-specific assessment and intervention to promote sexual health.

The CPG recommendations herein largely promote the following general best practices:

- Clinician self-evaluation and professional development to ensure competence in addressing sexuality throughout the lifespan without bias, judgement, or discrimination,
- A lifespan approach to sexuality promoting early developmental skills for independence and health management followed by ongoing formative evaluation and open communication during transition to and throughout adulthood to ensure early detection and intervention for at-risk, developing, or present impairments that may affect sexual health/participation,
- An education-based intervention model to promote self-awareness, health literacy, and informed personal decision making regarding medical and lifestyle-related sexual health choices.

At this time, there is not sufficient data to identify the efficacy and safety of most approaches to sexual health when applied to the EB population. Due to EB’s potential involvement of cutaneous and mucosal structures, genitourinary structures, and overall physical functioning, the efficacy and safety of typical mechanical methods of sexual health intervention, such as condoms and other physical barriers to prevent sexually transmitted diseases/infections, cannot be assumed generalizable to the EB population. The lack of EB-specific data on this and other lifesaving and health-preserving interventions related to sexuality presents a clear inequity in need of correction.

These guidelines provide an initial framework for supporting sexual health for people living with EB and seek to establish an open dialogue between the health care provider and the individual living with EB, as well as a larger dialogue within the EB community. To serve the community of people living with EB with equity, efficacy, and safety, further research is required.

## Further research

The authors of these guidelines acknowledge a lack of evidence in the literature to support strong recommendations. This panel has identified the following future research priorities based on the needs identified by this review and the EB community in initial survey responses:

- Data collection to improve understanding of frequency and nature of subtype-specific EB experiences of psychosocial and mechanical factors affecting sexuality and pubertal development,
- Standardization of methods and measures for assessing sexuality-related quality of life and needs among the EB population,
- Cultural perspectives/factors affecting experiences of sexuality within the EB population,
- Best practices for genetic counselling and education (timing, methodology, decision making),
- Specific assessment and intervention strategies for psychosocial factors affecting sexuality (self-image, body-image, confidence, etc.),
- Data informing the safety and efficacy of sexually transmitted disease/infection and contraceptive intervention strategies in the EB population,
- Assessment and intervention strategies relevant to EB child and adolescent psychosocial development, including data collection and education specifically related to pubertal maturation,
- Specific adaptations and modifications to address mechanical barriers to participation including but not limited to commercial and medical products/resources for positioning, lubrication/friction-reduction, maintenance of genitourinary structures, fatigue/pain reduction, and human and mechanical stimulation.

### Updating procedure and dissemination

The guidelines will be updated every 3–5 years or earlier if there is a significant breakthrough in EB sexuality health care treatment from the publication date. We recommend a literature search to see whether a full review is warranted at any stage.

DI aims to ensure that the EB CPG address the needs of patients internationally. The guidelines will be presented at the international DEBRA Congresses. This guideline has supplementary material which can be used as tools anywhere in the world. DI recommends that implementation of these recommendations should be monitored and evaluated through audits. The completion of a current practice audit, followed by the CPG pre-implementation survey (https://surveyhero.com/c/aabc0100) and post-implementation survey are highly recommended for best practice.

## Supplementary Information

**Additional file 1.** Clinical Resources for the Support of Sexuality.

**Additional file 2.** DI Clinical Practice Guideline Development Standard.

**Additional file 3.** Panel member’s affiliations and roles, Review panel affiliations.

**Additional file 4.** Limited results data from the scoping surveys completed by people living with EB.

## Data Availability

Not applicable

## Abbreviations

AGREE II: Appraisal of Guidelines for Research and Evaluation
CPG: Clinical Practice Guideline/s
DI: DEBRA International
EB: Epidermolysis bullosa
GRADE: Grading of Recommendations, Assessment, Development, and Evaluation
PPI: Patient population involvement
WHO: World Health Organization
