# Supplementary material for: Supporting sexuality for people living with epidermolysis bullosa: clinical practice guidelines
Source: Orphanet J Rare Dis. 2021 Jan 6;16:9. doi: 10.1186/s13023-020-01640-0 (PMC7789698; doi:10.1186/s13023-020-01640-0)
Supplement: Supplementary file 1 — Additional file 1. Clinical Resources for the Support of Sexuality. [file 13023_2020_1640_MOESM1_ESM.pdf]

Clinical Resources for the Support of Sexuality<br>Below is a list of widely available online and print resources to assist clinicians in meeting the expectations of this guideline. Clinicians are strongly encouraged to search for local support organizations and clarify local laws and regulations that affect boundaries of the clinical relationship and sexuality-related activities/freedoms.
**Physicians for Reproductive Health**<br>A collaborative effort to develop resources and advocate for access to comprehensive reproductive health care. Access support from experts, as well as as an extensive database of educational modules/presentation on best practices, LGBTQ essentials, sexual health, sexually transmitted infections, pregnancy, and other topics.<br>https://PRH.org; Provider education resources: https://prh.org/medical-education/
**World Health Organization – Human Reproduction Program**<br>Resources for sexual and reproductive health best practice resources and information, with an international and inclusive perspective.<br>https://www.who.int/reproductivehealth/en/
**National Coalition for Sexual Health**<br>Resources for providers and the public to promote communication and access to sexual health best practices.<br>https://nationalcoalitionforsexualhealth.org/
**Center on the Developing Child – Harvard University**<br>Resources for information and program development of evidence based developmental supports. This resource addresses psychosocial aspects of development including resilience and stress.<br>https://developingchild.harvard.edu/
**Body Matters: Interventions and change techniques designed to improve body image**<br>(Alleva, J.M. 2015)<br>Dissertation at Maastricht University – Evaluates the concept of body image and proposes evidence-based approaches to intervention.<br>Full Text Available:<br>https://cris.maastrichtuniversity.nl/en/publications/body-matters-interventions-and-change-techniques-designed-to-impr
**Sexualidad y Discapacidad**<br>[Spanish Language Resource]<br>(Rubio, Cruz, González, Celeda, Aparacio, & Fonfría, 2017)<br>Group promoting quality of life for people with disabilities with an emphasis on sexuality education and support.<br>http://sexualidadydiscapacidad.es/

Relevant print publication:<br>**Buenas Prácticas en Sexualidad y Enfermedades Raras**<br>[Best Practices in Sexuality and Rare Diseases]
**Pleasure ABLE: Sexual Device Manual for Persons with Disabilities**<br>(MacHattie, E., Naphtali, K. Disabilities Health Research Network 2009.)<br>A clinical and personal guide to types of commercially available products and resources to adapt sexual activities.<br>Full-Text and other resources available at: https://www.sexualitysci.org/resources
**Disability, Intimacy, and Sexual Health: A social work perspective**<br>(Linton, Rueda, & Williams 2017)<br>Research-based text exploring historical and contemporary perspectives and approaches to sexuality with an emphasis on promoting “full sexual citizenship of people with disabilities”<br>https://www.naswpress.org/publications/Health/disability-intimacy-and-sexual-health.html<br>ISBN: 978-0-87101-522-8
**DEBRA International**<br>“DEBRA International is the central body for a worldwide network of nearly 50 national DEBRA and EB patient support groups working on behalf of those affected by the genetic skin blistering condition epidermolysis bullosa (EB).”<br>Access to clinical practice guidelines, patient versions of CPG, and other education/research resources. Access to networking to connect providers supporting people living with EB.<br>https://www.debra-international.org/
**Sex & U**<br>From the Society of Obstetricians and Gynaecologists of Canada<br>Presents client-friendly information and resources for sexual and reproductive health.<br>https://www.sexandu.ca/
