# Supplementary material for: Supporting sexuality for people living with epidermolysis bullosa: clinical practice guidelines
Source: Orphanet J Rare Dis. 2021 Jan 6;16:9. doi: 10.1186/s13023-020-01640-0 (PMC7789698; doi:10.1186/s13023-020-01640-0)
Supplement: Supplementary file 2 — Additional file 2. DI Clinical Practice Guideline Development Standard. [file 13023_2020_1640_MOESM2_ESM.pdf]

Clinical Practice Guideline Development Standard

Steps	EB Clinical Practice Guidelines
1	**Establishment of panel and determination of clinical questions**<br><br>1. Select the clinical **topic** to focus on<br>2. Identify a clinical expert to be the **lead/chair** of the CPG (or engage a **project manager**)<br>3. Build the guideline development **panel**. The panel should:<br>• Include 8-12 individuals<br>• Include 2-3 patient representatives who should be involved in all steps of the guideline development and included as authors on the final publication.<br>• Be multidisciplinary, including experts in the clinical topic and experts in overlapping areas of clinical care.<br>• Include at least 3 different centres (and ideally countries) and more if possible.<br>• Ideally meet in person at least once (coordination with a DEBRA or EB-CLINET meeting might facilitate this). Online conferencing tools should be used for other meetings.<br>**Note**: people with valuable expertise, who are unable to be panellists can still be included through being asked to review the draft guideline. Any suggestions they make would need to be considered and agreed on by the panel, in a transparent fashion.<br>4. **Undertake preliminary literature search** and/or **audit of current practice** (this can support completion of the DEBRA application form and provide background information for the first panel meeting meeting)<br>5. Complete and **submit application form** to DEBRA international<br>6. **Scope out the population (patient) priorities** (this feeds into the first meeting and if completed prior to making the application it can be used as evidence here).<br>7. **Plan first panel meeting**<br>• Minimum of 6 members must be physically present for good group dynamics<br>• Other members can be linked through online conferencing tools<br>• Minutes should be taken and feedback requested from all panel members.<br>8. **Meeting plan:** group introductions (brief); panel ground rules (relating to communication, deadlines, responsibilities etc.); background on methodology to be adopted; presentation of preliminary data, presentation of patient priorities, determination of main clinical question(s) through use of the PICO (population, intervention, comparison and outcomes) framework; summary of meeting and allocation of jobs.<br>9. Rate clinical questions by importance and narrow down to 5-7<br>• Clinical Questions should be determined by **practice** (what do we

need to know) and **<u>NOT</u>** evidence driven
- Outcomes should be determined by **importance** to patients and **<u>NOT</u>** evidence driven

2

Systematic literature searches

The literature search should:
1. Assess guidelines (in the area or related area)
2. Be based on the prioritised 5-7 clinical outcomes
3. Follow a systemic system to ensure compatibility (in the case of more than one searcher) and that no data is missed
4. Involve sifting, selecting and removal of duplicates
5. Use more than 3 search engines
6. Possibly include trials registrations, conference abstracts, hospital protocols, other related guidelines
7. Be undertaken in different languages (other than just English).
8. Go as far back in date as possible in the case of a new guideline or back to date from when the last searches were conducted (or engines not previously used) in the case of a review.
9. Use separate searches for each clinical question

3

Systematic appraisal of papers identified in the search

The appraisal of papers should:
1. Assess the quality of the papers
2. Assess potential bias in the papers
3. Follow a systemic system to ensure compatibility (in the case of more than one appraiser) and that no data is missed
4. Involve each paper being appraised by at least 2 panel members to ensure consistency rating.
5. Involve a third member (lead, chair or project manager), where there is less than 50% consistency between appraisers.
6. Include all group study types for rare diseases: systemic reviews, meta-analysis, RCTs, cohorts studies, case control studies, observational studies and lastly expert opinions
7. Summarise the appraisal results by compiling an evidence profile for each question and study type

4

Formulation of recommendations

Plan final panel meeting
- Minimum of 6 members must be physically present for good group dynamics
- Other members can be linked through online conferencing tools
- Minutes should be taken and feedback requested from all panel members.

Meeting plan: group introductions (brief); panel ground rules (relating to communication, deadlines, responsibilities etc.); overview of plan for the meeting and decision framework to be adopted; report on literature search/appraisal; considered judgement of evidence, formulation of recommendations; drafting of

	recommendations (together with transparent explanations of how arrived at); summary of meeting and allocation of jobs.<br>Recommendations should be clear, transparent and actionable and use standard wording. They should include the:<br>• Direction of the recommendation (i.e. for or against)<br>• The strength of the recommendation<br>• The quality of the recommendation
5	**Writing and publication of guideline**<br><br>The final guideline should:<br>• Include a recommendations summary table where recommendations are clearly linked to evidence and transparency.<br>• Include all relevant information, according to the AGREE II tool<br>• Provide clear recommendations for further research in areas for which no evidence was identified<br>• Be sent for review by clinical and patient representatives who were not part of the panel (in order to bring fresh perspectives.)<br>• Incorporate reviewer feedback on the basis of agreement by the whole panel (with inclusion of footnotes where necessary for transparency)<br>• Be formatted as per journal instructions<br>• Be submitted for publication in an open access journal which permits a link to the full publication on the DEBRA international website

These standards was modified from the SIGN and GRADE methodologies.
