# Supplementary material for: Supporting sexuality for people living with epidermolysis bullosa: clinical practice guidelines
Source: Orphanet J Rare Dis. 2021 Jan 6;16:9. doi: 10.1186/s13023-020-01640-0 (PMC7789698; doi:10.1186/s13023-020-01640-0)
Supplement: Supplementary file 3 — Additional file 3. Panel member’s affiliations and roles, Review panel affiliations. [file 13023_2020_1640_MOESM3_ESM.pdf]

EB Sexuality Clinical Practice Guideline: Supplementary material 3

Panel member's affiliations and roles, Review panel affiliations

CPG working Panel

Panel member	Country of origin	Speciality with EB	Role in panel	Method and appraisal training	First meeting	Searches	Paper filtration and Appraisal	Outcome summaries	Recommendation meeting	Recommendation Summary	Writing	Panel Review	Incorporation of feedback from panel	Incorporation of feedback from reviewers	Submitting the CPG
Alex Ryan King	USA	Occupational Therapist lead of EB service for Paediatrics to young adults	Lead	✓	✓	✓	✓	✓	✓	✓	✓	✓	✓	✓	✓
Humphrey Hanley	New Zealand	PPI/ RDEB/ DEBRA New Zealand	Co-lead				✓	✓	✓	✓	✓	✓	✓	✓	✓
Mark Popenhagen	USA	Psychologist Specializing in EB and Pain/ and Guideline methodologist	Member			✓	✓	✓	✓	✓		✓	✓	✓	
Florencia Perez	Chile	Comminuty Psychologist	Member		✓	✓	✓	✓	✓	✓		✓		✓	
Kerry Thompson	Australia	PPI/ DDEB/ A&E nurse specialist	Member		✓	✓	✓	✓	✓	✓		✓		✓	
Diana Purvis	New Zealand	Paediatric and Adolescent Dermatologist/ reported CoI	Member			✓	✓	✓				✓	×	×	
Nora Garcia Garcia	Spain	Comminuty Psychologist	Member		✓							✓			
Ida Steinlein	Norway	PPI/IRDEB/ DEBRA Norway	Member		✓							✓			
Mia Werkentoft	Sweden	PPI/JEB/ DEBRA Sweden	Member		✓							✓			
Matthew Lightfoot	UK	PPI/ DDEB/ DEBRA UK	Member						✓	✓		✓		✓	
Michelle Lahat	USA	Clinical Social Worker II	Member			✓	✓	✓	✓	✓		✓		✓	
Kalsoom Begum	UK	Adult CNS	Member				✓	✓				✓			
Julio Tanabe	USA	PPI/ RDEB	Member			✓			✓	✓					

Key: UK- United Kingdom; USA- United States of America; EB- Epidermolysis bullosa; CNS- EB Clinical Nurse Specialist; DI- DEBRA International; PPI- Patient and Public involved (People living with EB); CoI- Conflict of interest; ✓- involved in this development step; ×- not involved in this development step due to CoI.

Reviewer Panel List

Angela Roberts	South Africa	PPI	DEBRA South Africa
Anna Carolina Rocha	Brazil	PPI	DEBRA Brazil
Caroline Francis	UK	PPI	DEBRA UK
Catherine Hayes	UK	Professor and Reader in Health Professions Pedagogic	University of Sunderland
Catherine McCuaig	Canada	Professor of Dermatology in Pediatrics	Université de Montréal
Emma King	Australia	EB Nurse Practitioner	Melbourne Childrens Hospital
Maria Florencia Fernandez	Argentina	Paediatric Dermatologist	DEBRA Argentina
Natalie Yerlett	UK	Specialist EB Paediatric Dietitian	GOSH
Sarah Morrill	USA	PPI	
Sofia Larrain Moore	Chile	Physiotherapist	DEBRA Chile
Tracey Vlahovic	USA	Clinical Professor Department of Podiatric Medicine	Temple University
Yitka Graham	UK	Head of Helen McCargle Nursing Research and Care	University of Sunderland
Zena Moore	Ireland	Professor and Head of the School of Nursing and	RCSI

Key: UK- United Kingdom; EB- Epidermolysis bullosa; CNS- EB Clinical Nurse Specialist; GOSH- Great Ormond Street Hospital; RCSI- Royal College of Surgeons in Ireland; CoI- Conflict of interest;- ✓ Expert presenter at the First panle meeting; *Panel members who has to resign their roles due to work commitmnets and requested to support through the review process.
