# Supplementary material for: Supporting sexuality for people living with epidermolysis bullosa: clinical practice guidelines
Source: Orphanet J Rare Dis. 2021 Jan 6;16:9. doi: 10.1186/s13023-020-01640-0 (PMC7789698; doi:10.1186/s13023-020-01640-0)
Supplement: Supplementary file 4 — Additional file 4. Limited results data from the scoping surveys completed by people living with EB. [file 13023_2020_1640_MOESM4_ESM.pdf]

Limited results data from the scoping surveys completed by people living with EB

As no significant data of this nature or volume was identified in the published literature during the CPG process, the following has been provided to guide discussions of research and clinical practice for addressing topics of sexuality for people living with Epidermolysis Bullosa.

The below descriptive data represents responses of people living with EB in the initial scoping surveys for the Sexuality and EB CPG. Demographic and otherwise potentially identifying survey items have been removed and written comments excluded or summarized to protect the privacy of the respondents. All respondents to these surveys were informed in writing prior to survey completion that participation was voluntary and carried no expectation of compensation. All respondents consented to the use of any information provided in the guideline process.

While specific demographics have been removed, it is important to note that the respondent group is representative of all 4 major subtypes of EB, adolescent through older adult age groups, 18 countries, all levels of education (less than secondary school to doctoral levels), 13 religious/spiritual affiliations, 2 genders (86% female, 14% male), and 5 sexual orientation groups (majority heterosexual at 84%).

What types of sexual experiences have you had?

Number of responses: 88

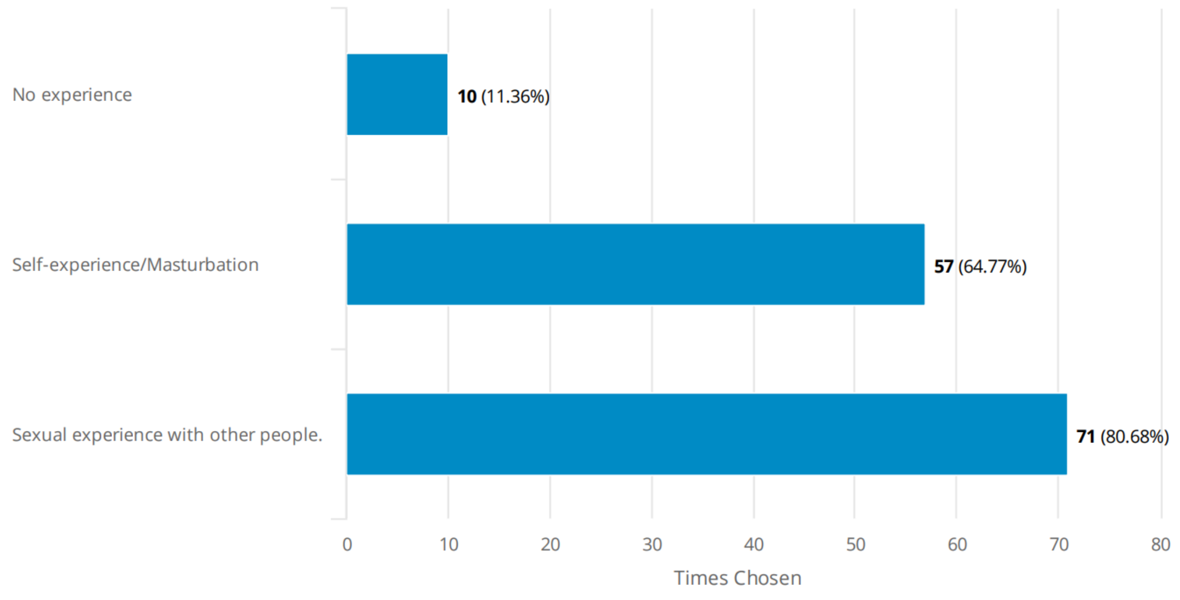

Overall, how satisfied are you with your sexual lifestyle?

Number of responses: 88

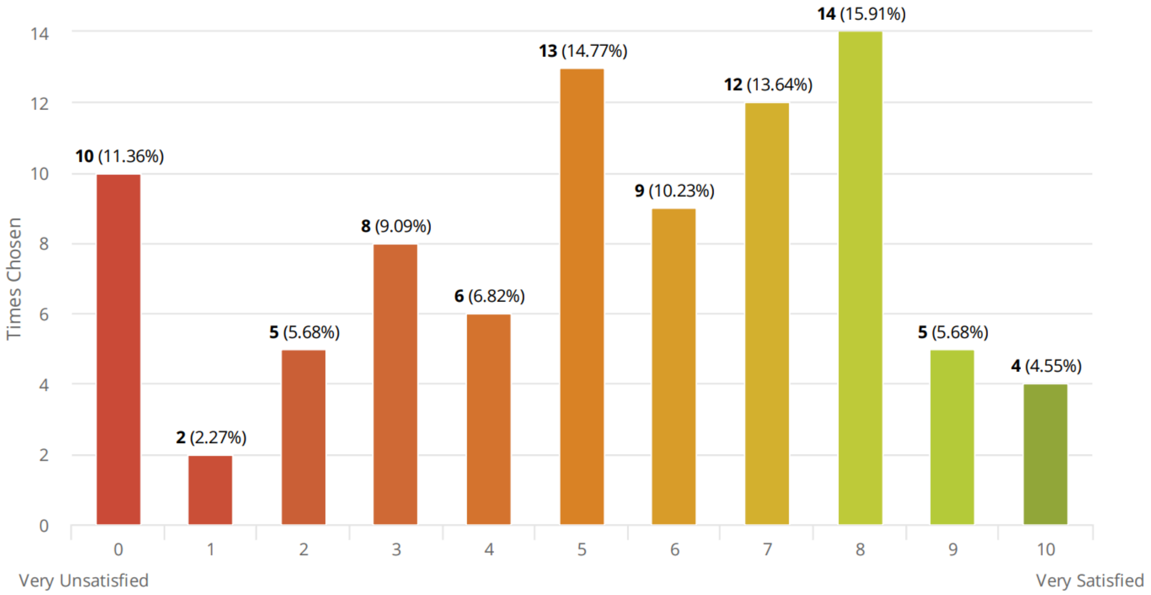

How much do you feel your EB influences ability to find/start an intimate relationship?

Number of responses: 88

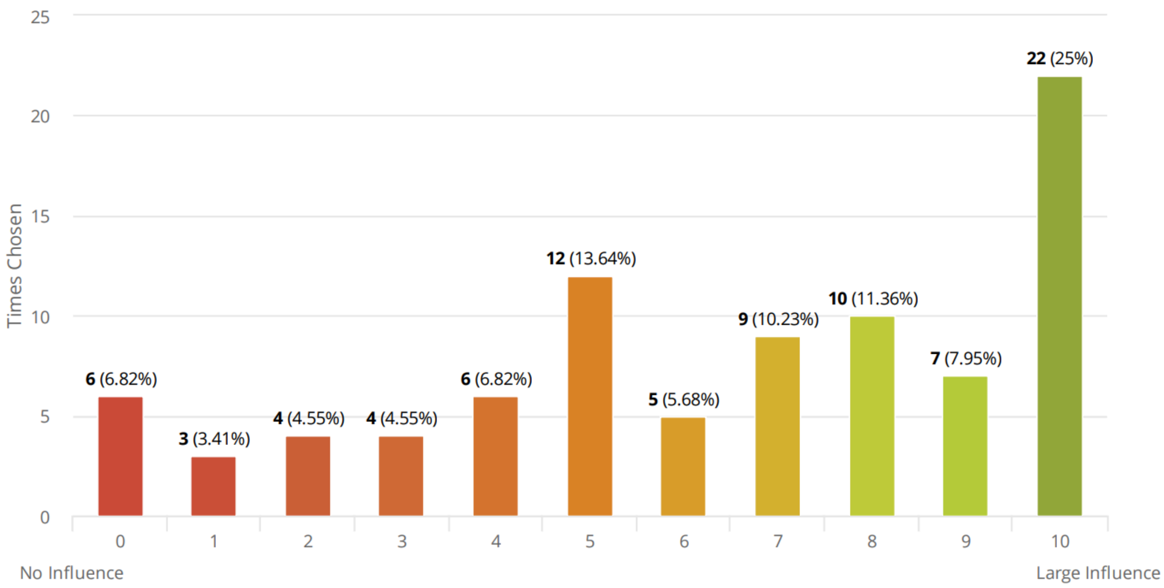

How much do you feel your EB influences your ability to maintain/keep an intimate relationship?

Number of responses: 86

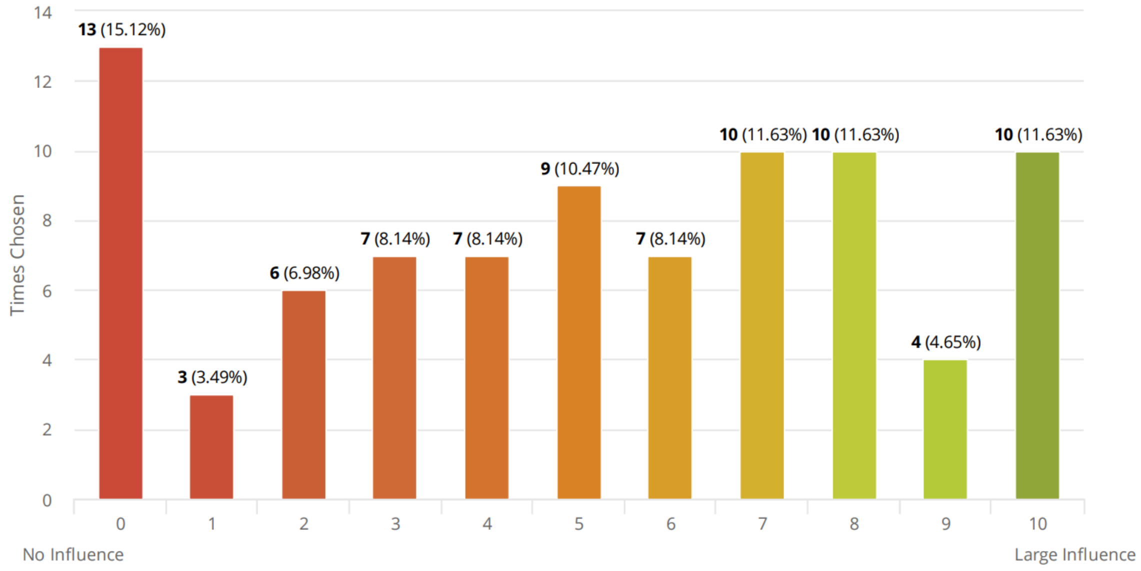

How much does your EB affect your ability to enjoy sexual experiences?

Number of responses: 86

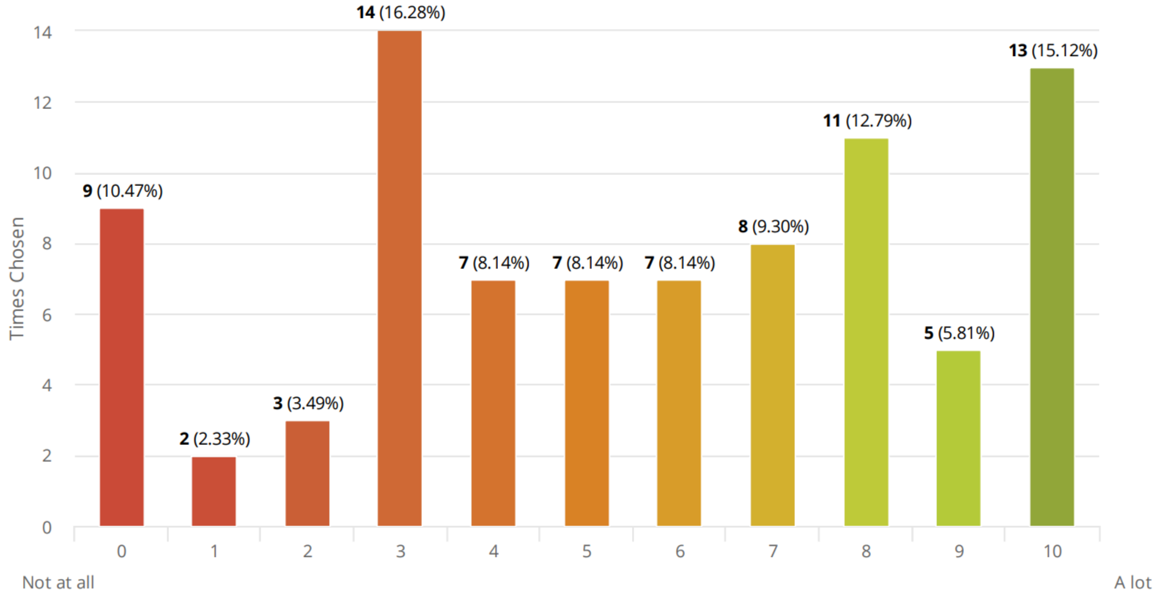

How much does your EB affect your partner's ability to enjoy sexual experiences?

Number of responses: 85

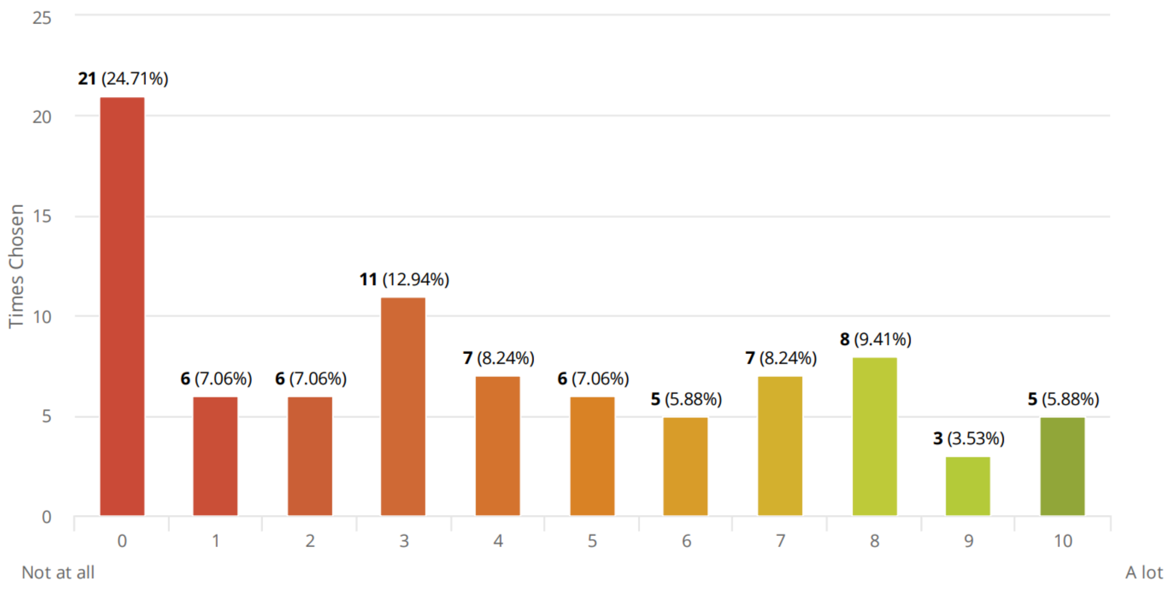

What, if any, EB-related barriers have you experienced related to sexual/intimate relationships/activities.

Number of responses: 87

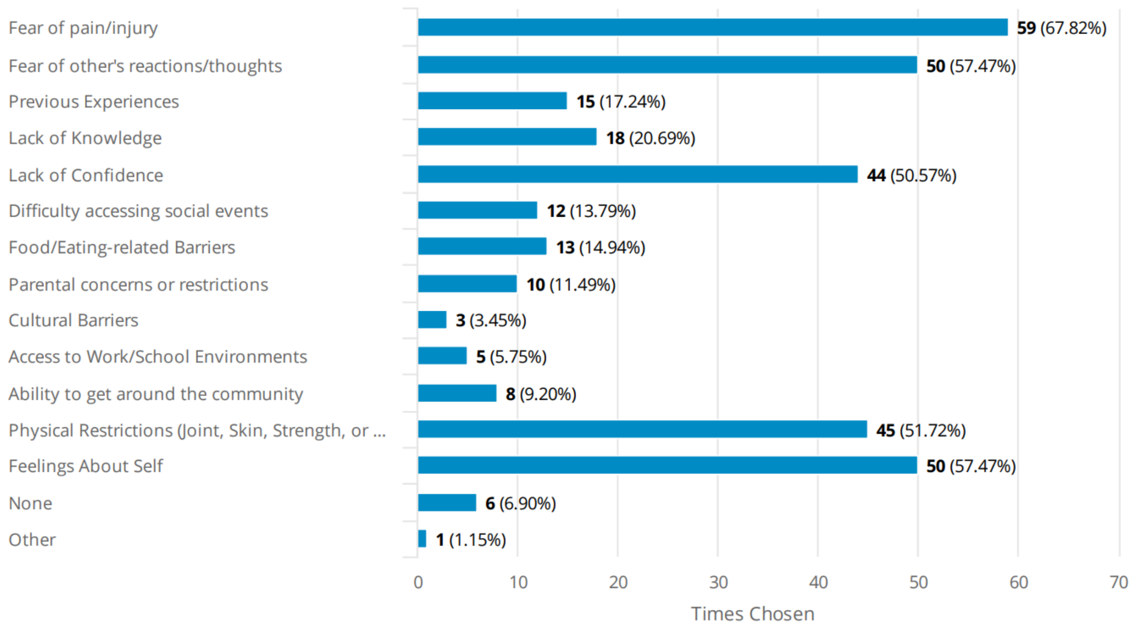

"Other" response indicated lack of partner understanding in comment section.

What, if any, strategies or adaptations have helped increase your ability to access sexual/intimate relationships/activities?

Number of responses: 84

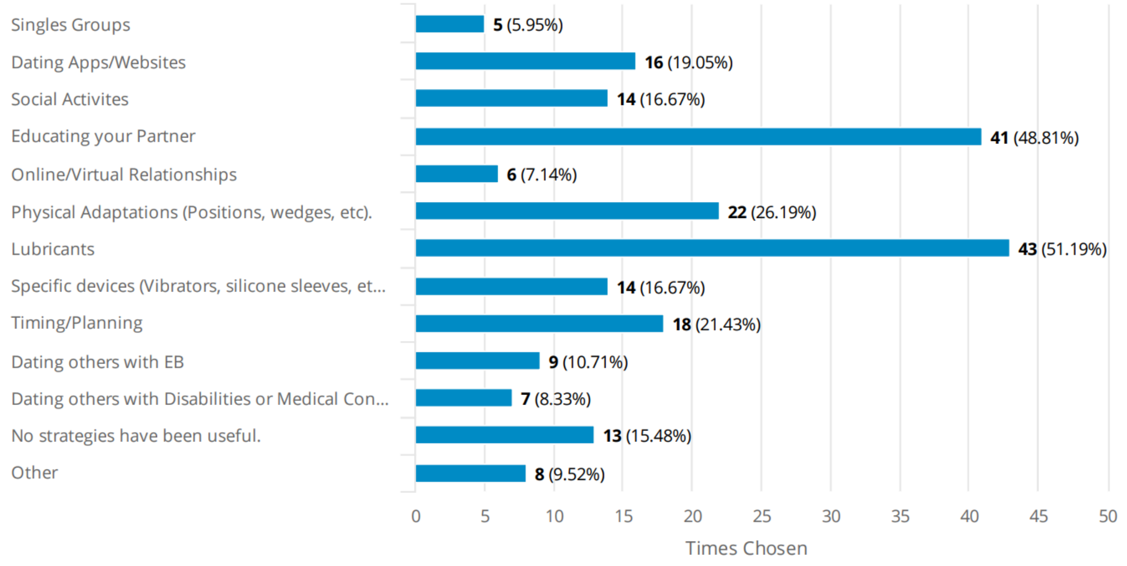

"Other" responses indicated importance of self-knowledge, confidence, and communication skills. Foreplay was also mentioned as a valued strategy.

Select the 5 most important areas of focus for the EB Clinical Practice Guideline on Sexuality:

Number of responses: 82

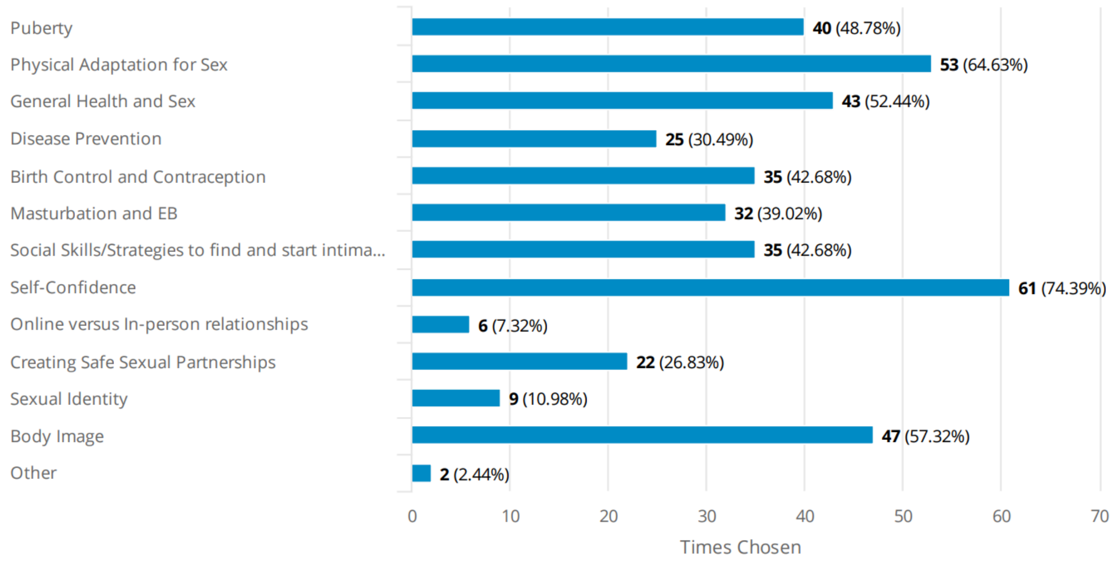

"Other" responses indicated importance of pain management in sexual activities, as well as early and ongoing management of EB involvement in genitals and sexual organs to maintain the ability to participate in sex.

Additional comments provided by respondents included too much identifying information to include in full form, however the following topics were introduced or emphasized:

- Individual barriers:
  - Fear of pain and/or injury,
  - Body image,
  - Self-esteem, self-worth, confidence, and self-efficacy,
  - Concern about perception of others (partners and potential partners),
  - Hand and mouth involvement affecting valued sexual activities,
  - EB involvement of genitals/sexual organs limiting sexual participation,

- Importance of strategies/adaptations:
  - Importance of lubrication and friction limiting strategies,
  - Importance of effective positioning for sexual activities,
  - Managing duration of sexual activities,
  - Managing dressings/bandages,
  - Strong communication with partners;

- System/Community Level Barriers:
  - The right to sexuality and the barrier created by stigma around sex and disability,
  - Stigma around masturbation for men and women,
  - Lack of accessible information about EB and sexuality in community and healthcare settings,
  - Lack of LGBTQ+ awareness and resources among healthcare providers,
  - Lack of information regarding contraception options and sexually transmitted infection prevention.
